# TAK1 Binding Protein 2 Is Essential for Liver Protection from Stressors

**DOI:** 10.1371/journal.pone.0088037

**Published:** 2014-02-03

**Authors:** Yuka Ikeda, Sho Morioka, Kunihiro Matsumoto, Jun Ninomiya-Tsuji

**Affiliations:** 1 Department of Biological Sciences, North Carolina State University, Raleigh, North Carolina, United States of America; 2 Department of Molecular Biology, Graduate School of Science, Nagoya University, Nagoya, Japan; Univeristy of California Riverside, United States of America

## Abstract

The liver is the first line of defense from environmental stressors in that hepatocytes respond to and metabolize them. Hence, hepatocytes can be damaged by stressors. Protection against hepatic cell damage and cell death is important for liver function and homeostasis. TAK1 (MAP3K7) is an intermediate of stressors such as bacterial moieties–induced signal transduction pathways in several cell types. Tak1 deficiency has been reported to induce spontaneous hepatocellular carcinoma. However, the regulatory mechanism of TAK1 activity in liver stress response has not yet been defined. Here we report that activation of TAK1 through TAK1 binding protein 2 (TAB2) is required for liver protection from stressors. We found that a bacterial moiety, lipopolysaccharides (LPS), activated TAK1 in primary hepatocytes, which was diminished by deletion of TAB2. Mice having hepatocyte-specific deletion of the Tab2 gene exhibited only late-onset moderate liver lesions but were hypersensitive to LPS-induced liver injury. Furthermore, we show that a chemical stressor induced greatly exaggerated liver injury in hepatocyte-specific Tab2-deficient mice. These results demonstrate that TAB2 is a sensor of stress conditions in the liver and functions to protect the liver by activating the TAK1 pathway.

## Introduction

The liver is responsible for the first pass metabolism of absorbed exogenous chemicals and the major organ of clearance of toxicants and pathogens, and liver cells are constantly exposed to those foreign substances including bacterial moieties and food- and clinical drug-derived chemicals. These stressors directly impact on liver parenchymal cells, and indirectly through cytokines from activated circulating and residential immune cells such as Kupffer cells [1], [2]. Although the stressors and stressor-induced cytokines could activate cell death signaling in hepatocytes, they are normally resistant to those basal levels of stressors and functional under the homeostatic conditions. Excessive stressors derived from alcohol consumption and pathogens lead to liver injury. Furthermore, dysregulation of the protecting responses is likely to be associated with liver tumors in which cell death causes compensatory cell proliferation [3]. However, the mechanism by which hepatocytes respond to stressors and protect against cell death is still largely elusive.

Transcription factor NF-κB and mitogen-activated protein kinase, JNK, have been implicated in the regulatory mechanisms of hepatocyte death [4], [5]. NF-κB protects hepatocytes from concanavalin A-induced cell death, and ablation of NF-κB induces spontaneous hepatocyte death and compensatory proliferation [4], [6]. Activation of JNK is also closely associated with liver injury [5], [7], [8]. NF-κB transcriptionally activates several cell survival genes such as caspase inhibitors, cellular FLICE-like inhibitory protein (cFLIPL) and cellular inhibitor of apoptosis proteins (cIAPs), and anti-oxidant genes such as glutathione peroxidase and superoxide dismutase, which block proinflammatory cytokine TNF-induced cell death and oxidative damage [4], [9]-[11].

TAK1 (MAP3K7) is a member of the mitogen-activated protein kinase kinase kinase (MAP3K) family and an intermediate of stress-induced intracellular signaling pathways [12]–[14]. TAK1 activating stressors include proinflammatory cytokines TNF and IL-1 and Toll-like receptor ligands as well as chemical and physical stress, such as osmotic stress. TAK1 is the major upstream signaling mediator leading to activation of NF-κB and upregulation of antioxidant enzymes, thereby participating in cell survival in several tissues *in vivo* [15], [16]. Ablation of TAK1 causes cell death associated with oxidative damage and tissue injury in the epidermis, the intestinal epithelium, and blood vessels [16]–[18]. The major cause of cell death in *Tak1*-deficient tissues *in vivo* is TNF-induced cell death, and deletion of TNF receptor 1 largely rescues cell death and tissue injury in these *Tak1*-deficient tissues [16], [18]. Similar to these tissues, hepatocyte-specific deletion of the *Tak1* gene causes cell death and liver injury, which further causes compensatory hepatocyte proliferation resulting in development of hepatocellular carcinoma [19], [20]. Thus, TAK1-mediated cell signaling presumably of NF-κB and antioxidant gene regulation is indispensable for hepatocyte protection from stressor-induced cell death. However, the pathway through which TAK1 is activated in hepatocytes *in vivo* has not yet been determined.

TAK1 binding partner proteins, TAK1 binding proteins 1, 2 and 3 (TAB1, TAB2 and TAB3), mediate activation of TAK1 through two different mechanisms. TAB1 mediates TAK1 oligomerization to induce autophosphorylation of TAK1 within the activation loop in the protein kinase domain, which catalytically activates TAK1 [13], [21], [22]. TAB2 and TAB3 are related proteins, both of which bind to polyubiquitin chains and recruit TAK1 to the polyubiquitin chain protein complexes [23]–[27]. Polyubiquitin-mediated oligomerization of TAK1 proteins facilitates TAK1 protein kinase activation. The TAB1-dependent mechanism is required for osmotic stress-induced TAK1 activation [13] and the basal TAK1 activity in the epidermis *in vivo* [28]. The polyubiquitin-mediated mechanism activates TAK1 in response to TNF, IL-1 and TLR ligands. Although both TAB2 and TAB3 can mediate the polyubiquitin mechanism, TAB2 plays a predominant role in TAK1 activation in several cell types including endothelial and immune cells [17], [23]. Double deficiency of *Tab1* and *Tab2* abolished TAK1 activity in the epidermis in vivo, which caused TNF-induced cell death and tissue damage resembling the phenotypes of TAK1 deficiency [28]. However, single deletion of either *Tab1* or *Tab2* does not cause any abnormalities in the epidermis, demonstrating that TAB1 and TAB2 are functionally redundant in the epidermis. Overall, TAB1 and TAB2 are major activators of TAK1 in several cell types, and contributions of TAB1- and TAB2-dependent mechanisms to TAK1 activity vary depending on cell types. In the current study, we investigated the role of TAB2 in hepatocytes and determined that TAB2 activates TAK1 in response to a TLR ligand lipopolysaccharide (LPS), which is essential for hepatocyte survival.

## Materials and Methods

### Mice

*Tak1*-floxed (*Tak1^flox/flox^*), *Tab2*-floxed (*Tab2^flox/flox^*), *Tab1*-floxed (*Tab1flox/flox*), mice were previously described [29]–[31]. *Rosa26-CreERT* [32] and *Alb-Cre* [33] transgenic mice were obtained from the Jackson Laboratories. All strains used were on a C57BL/6 background. Induction of *Tab2* deletion in *Rosa-CreER Tab2^flox/flox^* was achieved by intraperitoneal (ip) injection of tamoxifen (50 mg/kg mouse weight) for 3 consecutive days. The tamoxifen injected mice were used for the experiments 3–6 weeks post injection to avoid potential acute toxicity derived from the transient Cre expression as described previously [34]. All animal experiments were conducted with the approval of the North Carolina State University Institutional Animal Care and Use Committee (IACUC protocol # 11-138B). All efforts were made to minimize animal suffering.

### Reagents

Reagents used were tamoxifen (MP Biomedicals) and 4-hydroxytamoxifen (Sigma) LPS (From *Salmonella minnesota*, phenol extraction, Sigma) and diethylnitrosamine (DEN) (Sigma). Polyclonal antibodies used were TAK1, TAB1, and TAB2 described previously [14], [35], phosphorylated TAK1, cleaved caspase 3, phosphorylated JNK, JNK (Cell signaling), β-actin (Sigma-Aldrich), p65 and α-tubulin (Santa Cruz). An ALT assay kit (Biovision, Mountain View, CA) was used.

### Primary Hepatocyte Culture

Primary hepatocytes were isolated with a standard collagenase procedure according to the instructions of Amaxa Biosystems (Amaxa, Gaithersburg, MD). Hepatocyte viability was assessed by the trypan blue dye assay, and only hepatocytes with 90% and above viability were used. Hepatocytes were plated on collagen-coated 60-mm dishes for protein isolation or 12-well plates for caspase 3 assay. Since *Tak1* deficiency severely damages the liver even within one month of mouse age, we utilized an inducible deletion system to prepare *Tak1^+/+^*, *Tak1* ^Δ/Δ^, *Tab1Tab2^+/+^* and *Tab1Tab2^DKO^* hepatocytes. *Rosa-CreER Tak1^flox/flox^*, *Rosa-CreER Tab1^flox/flox^Tab2^flox/flox^* hepatocytes were immediately treated with 0.1 µM 4-hydroxytamoxifen or vehicle (ethanol), and the medium containing 4-hydroxytamoxifen or vehicle was replaced every 24 h for 72 h and subsequently stimulated with 1 µg/ml LPS. To prepare Tab2-deficient hepatocytes, we used hepatocyte-specific *Tab2*-deficient and control littermate mice. *Alb-Cre Tab2^flox/flox^ (Tab2^-/-^)* and *Tab2^flox/flox^ (Tab2^+/+^)* hepatocytes were cultured overnight and simulated with 1 µg/ml LPS.

### Quantitative Real Time PCR Analysis

Total RNA was isolated from the liver using an RNeasy kit (Qiagen) and transcribed into cDNA using SuperScript VILO cDNA Synthesis Kit (Life Technologies). Expression levels of *Tak1* and *Tab2* were determined by quantitative real time PCR (qPCR) and normalized to the level of *Gapdh*. The following primers were used: *Tab2*-forward, 5′-GGATAGAATAAGCGAAGCCCGGAA-3′; *Tab2*-reverse, 5′-CTCTTTGAAGCCGTTCCATCCT-3′; *Gapdh*-forward, 5′-GAAGGTCGCTGTGAACGGA-3′; and *Gapdh*-forward, 5′-GTTAGTGGGGTCTCGCTCCT-3′. Primers for the Collagen type1 α1 *(Col1a1)*, tissue inhibitor of metalloproteinase 1 *(Timp1)* and H19 genes were generated according to a previous report [19]. *Cxcl2* and *Ccl2* mRNAs were detected by using the TaqMan® system (Life Technologies)

### Immunoblotting

Cell extracts were prepared using a lysis buffer (20 mM HEPES (pH 7.4), 150 mM NaCl, 12.5 mM β-glycerophosphate, 100 nM calyculin A, 1.5 mM MgCl_2_, 2 mM EGTA, 10 mM NaF, 2 mM DTT, 1 mM Na_3_VO_4_, 1 mM phenylmethylsulfonyl fluoride, 20 µM aprotinin, and 0.5% Triton X-100). Liver extracts were prepared in the abovementioned lysis buffer containing a protease inhibitor cocktail (G-Biosciences, St Louis, MO). Proteins were resolved on SDS-PAGE and transferred to Hybond-ECL or Hybond-P membranes (GE Healthcare). The membranes were immunoblotted with various antibodies, and the bound antibodies were visualized with horseradish peroxidase-conjugated antibodies against rabbit or mouse IgG using the ECL or ECL advance Western blotting detection kit (GE Healthcare).

### Cell Death Assays

Terminal dUTP nick-end labeling (TUNEL) assay was performed on formalin-fixed paraffin sections using an apoptotic cell death detection kit (Promega) according to the manufacturer's instructions. Seven to ten immunofluorescent images per mouse were randomly photographed, and at least 2000 DAPI-stained cells per mouse were counted. Quantitative results were generated from the counted numbers in 3-4 mice from independent experiments. 50 µg proteins from liver or hepatocyte extracts were used for Caspase 3 assay (Promega).

### Electrophoresis Mobility Shift Assay (EMSA)

The binding mixture contained radiolabeled ^32^P-NF-κB oligonucleotide probe (Promega), 10 µg of cell extracts, 4% glycerol, 1 mM MgCl_2_, 0.5 mM EDTA, 0.5 mM DTT, 50 mM NaCl, 10 mM Tris-HCl (pH 7.5), 500 ng of poly (dI-dC) (GE Healthcare), and 10 µg of bovine serum albumin to a final volume of 15 µl. The reaction mixture was incubated at 25°C for 30 min, separated by 5% (w/v) polyacrylamide gel, and visualized by autoradiography.

### Statistical Analysis

Results are expressed as either mean ± standard error of the mean (SEM) or mean ± standard deviation (SD). Differences between two groups were determined by two-tailed unpaired Student t test. Probability values of P are shown in figures. P≤0.05 was considered statistically significant.

## Results

### LPS Activates TAK1 through TAB2 in Primary Hepatocytes

**Figure 1:**
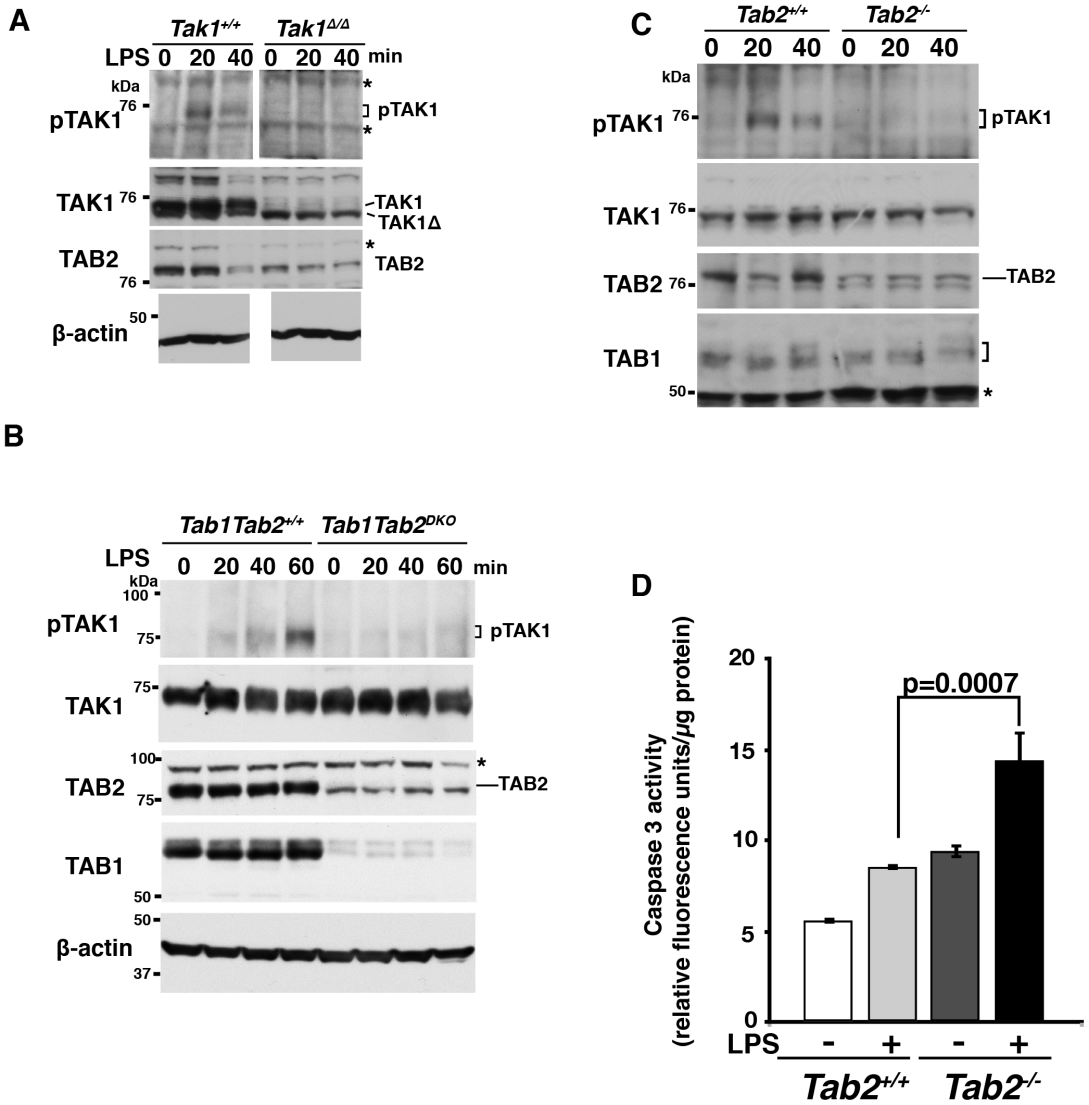
LPS activates TAK1 through TAB2 in primary hepatocytes. *Tak1*- (A), *Tab1* and *Tab2* double- (B) or *Tab2*- (C and D) deficient and control primary hepatocytes were prepared as described in Material and Methods, and stimulated with 1 µg/ml LPS. Cell lysates were analyzed by immunoblotting. In these *Tak1*-floxed mice, Cre-mediated recombination resulted in deletion of 38 amino acids of TAK1, and the truncated TAK1 (TAK1Δ) was expressed at a low level presumably due to reduced protein stability as indicated in A. Asterisks indicate non-specific bands. (D) *Tab2*-deficient and control primary hepatocytes were stimulated with 1 µg/ml LPS for 24 h. Cell lysates were subjected to caspase 3 assay. Data are shown as means ± SD, n = 3.

Ablation of TAK1 in hepatocytes is known to spontaneously induce apoptosis in mouse liver (19,20). We hypothesized that TAK1 is activated through TAB1- and/or TAB2-dependent mechanisms by stressors and prevents stressor-induced apoptosis in the liver. To begin to test this hypothesis, we investigated whether stressors can activate TAK1 in hepatocytes. We found that LPS activates TAK1 in primary hepatocytes. TAK1 was activated and the activity peaked at 20–60 min following LPS challenge in primary hepatocytes (Fig. 1A). To determine whether TAB1 and/or TAB2 participate in LPS-induced TAK1 activation, we utilized primary hepatocytes having double deficiency of *Tab1* and *Tab2*, and examined LPS-induced TAK1 activation. *Tab1* and *Tab2* double-deficient primary hepatocytes failed to activate TAK1 following LPS stimulation (Fig. 1B), demonstrating that TAB1- and/or TAB2-dependent mechanisms participate in the LPS signaling pathway. Furthermore, we found that *Tab2* single deficient primary hepatocytes failed to activate TAK1 following LPS stimulation (Fig. 1C). The time course and the level of TAK1 activation upon LPS challenge were slightly varied on each primary preparation, but activation of TAK1 was always detected in wild type hepatocytes. In contrast, *Tak1*-, *Tab1/Tab2* double- or *Tab2* single-deficient hepatocytes did not exhibit any increase of TAK1 activity in all preparations. These results suggest that a bacterial moiety LPS activates TAK1 through the TAB2-dependent mechanism in hepatocytes. We also examined whether NF-κB activation, which is a well-known downstream target of TAK1, is altered by *Tak1* or *Tab2* single deletion in hepatocytes. We found that *Tak1* deletion moderately reduced LPS-induced NF-κB activation in hepatocytes, suggesting that LPS activates NF-κB through multiple pathways including TAK1 pathway in hepatocytes (Fig. S1A). Similarly, *Tab2* deletion partially reduced LPS-induced NF-κB activation (Fig. S1B). These results demonstrate that TAB2 is likely to function as an activator of TAK1 in response to LPS in hepatocytes, which may be important for prevention of liver injury. The LPS-TLR4 pathway has been reported to engage apoptosis and necrosis [36]–[38]. To begin to determine whether TAB2 is important for liver integrity, we asked whether deletion of *Tab2* enhances LPS-induced cell death signaling. We examined caspase activity in LPS-treated *Tab2*-deficient primary hepatocytes. *Tab2*-deficient hepatocytes possessed slightly higher activity of caspase 3 compared to littermate control hepatocytes under unchallenged conditions, and caspase 3 activity was moderately but significantly upregulated by LPS treatment in *Tab2*-deficient hepatocytes (Fig. 1D). However, we did not observe any detectable increase in cell death in both wild type and *Tab2*-deficient hepatocytes following LPS stimulation. The weak activation of caspase 3 may be insufficient to induce cell death in *Tab2*-deficient hepatocytes. Thus, TAB2-dependent TAK1 activation is at least preventive to LPS-induced caspase 3 activation, and LPS is not a strong inducer of cell death in cultured hepatocytes. However, LPS is a strong inducer of liver injury *in vivo*, which is mediated not only through its direct effect on hepatocytes but through indirect stress through inflammatory responses. We speculate that that TAB2-dependent activation of TAK1 may be important for prevention of LPS-induced liver injury *in vivo*.

### TAB2 Knockout Mice Are Hypersensitive to LPS-Induced Liver Injury

**Figure 2:**
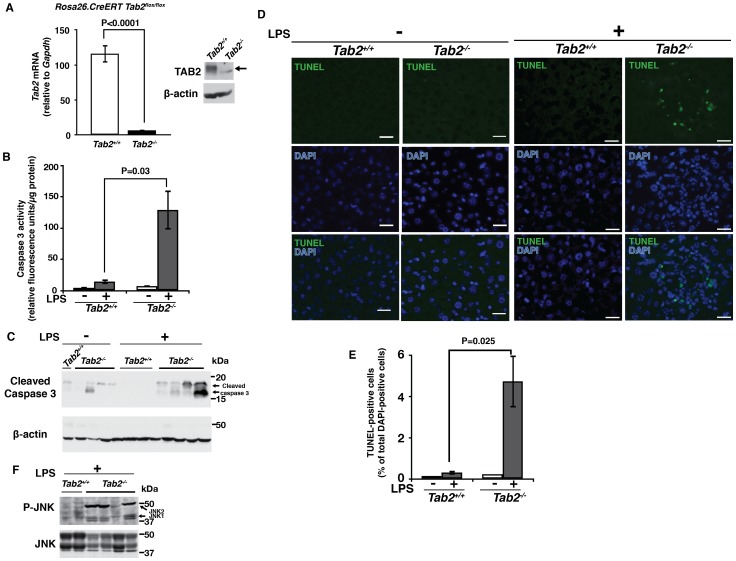
LPS induces liver damage in Tab2-deficient mice. (A–C) Adult *Tab2^+/+^* and *Tab2^-/-^* mice were generated from *Tab2^flox/flox^* and *Rosa-CreER Tab2^flox/flox^* mice by ip injection of tamoxifen as described in Experimental Procedures. (A) *Tab2* expression in the liver was determined by real-time PCR and immunoblotting at more than 3 weeks after tamoxifen injection. *Tab2^+/+^* mice n = 5; *Tab2^-/-^* mice n = 6. Means ± SEM and P values are shown. β-actin is shown as a loading control. Arrow indicates the position of TAB2. (B, C) *Tab2^-/^* ^-^ and control littermate or age matched mice were treated by ip injection of 8 mg/kg mouse weight LPS or vehicle (PBS) for 6 h, and the liver was isolated and protein extracts were subjected to caspase 3 assay (B) and analyzed by immunoblotting with anti-cleaved caspase 3 (C). Number of mice; untreated mice n = 3; LPS-treated *Tab2^+/+^* mice n = 4, *Tab2^-/-^* mice n = 4. Means ± SEM and P values are shown (B). (D, E) Liver sections of samples described in B and C were analyzed by TUNEL staining (D). Scale bars, 20 µm. TUNEL-positive cells per more than 2000 total liver cells per mouse were counted (E). Number of mice; untreated mice n = 2; LPS-treated *Tab2^+/+^* mice n = 3, *Tab2^-/-^* mice n = 3. Means ± SEM and P values are shown. (F) Liver proteins described in B and C were analyzed by immunoblotting with anti-phospho-JNK and anti-JNK. Each lane in C and F represents proteins from one animal.

To examine whether TAB2 plays a protective role in the liver *in vivo*, we generated TAB2^-/-^ adult mice by using a ubiquitously expressing inducible Cre deleter strain, *Rosa26.CreERT* [32]. *Tab2* deletion was induced by intraperitoneal injection of the CreERT activator, tamoxifen, and *Tab2* mRNA and protein were greatly diminished at 3 weeks after tamoxifen injection (Fig. 2A). Although germline deletion of *Tab2* is known to cause embryonic lethality around embryonic days 13-14 due to liver degeneration [31], *Tab2* deletion in adult mice did not cause overt liver abnormalities. This suggests that TAB2 plays an indispensable role during embryogenesis which is not required for adult liver homeostasis at least under unchallenged conditions. We asked whether *Tab2* deletion sensitizes the liver to LPS-induced injury in adult mice. *Tab2* deletion alone did not induce cell death in the liver, but LPS challenge greatly upregulated caspase activity in *Tab2*-deficient liver but not in the control within 6 h (Figs. 2B and 2C). TUNEL-positive cells with LPS challenge were highly increased by *Tab2*-deficiency (Figs. 2D and 2E). JNK activity was upregulated in *Tab2*-deficient liver (Fig. 2F), which is known to be associated with liver damage [8] and has been also observed in *Tak1*-deficient liver in earlier studies [19], [20]. We note that, because *Tab2*-deficient liver was greatly damaged within 6 h post-LPS injection, we were not able to analyze the consequences beyond 6 h. These results demonstrate that TAB2 is indispensable in the protection of the liver from LPS-induced cell death.

### Hepatocyte-Specific Deletion of Tab2 Induces Late Onset Liver Damage and Sensitizes Mice to LPS-Induced Liver Injury

**Figure 3:**
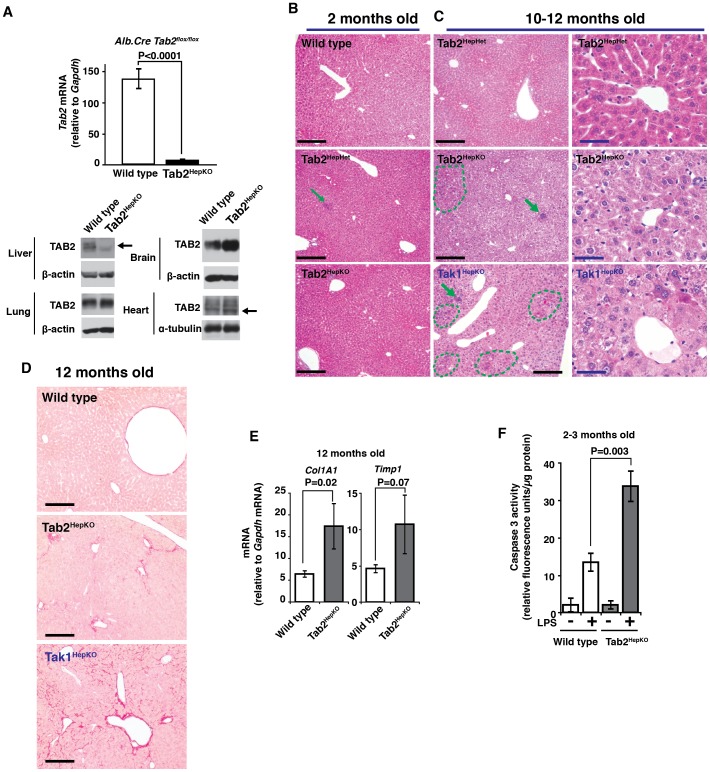
Hepatocyte-specific deletion of *Tab2* induces late-onset fibrosis and sensitizes the liver to LPS-induced injury. (A) *Tab2* expression in the liver, lung, brain, and heart was determined by real-time PCR (liver) and immunoblotting (liver, lung, brain and heart) in hepatocyte-specific *Tab2*-deficient, *Alb-Cre Tab2^flox/flox^* (Tab2^HepKO^), and littermate or age matched control, *Tab2^flox/flox^* (wild type), mice. Wild type mice, n = 10; Tab2^HepKO^ mice, n = 8. Means ± SEM and P values are shown. β-actin or α-tubulin is shown as a loading control. Arrows indicate the position of TAB2. (B) Hematoxylin and eosin staining of 2-month-old controls, including wild type and Tab2 heterozygous deletion, *Alb-Cre Tab2^flox/+^* (Tab2^HepHet^), and Tab2^HepKO^ liver sections. Scale bars, 200 µm. (C) Liver sections of 10- to 12-month-old control Tab2^HepHet^, Tab2^HepKO^ and Tak1-deficient (Tak1^HepKO^) mice. Left panels show overall structure of liver. Arrows indicate immune cell infiltration, and dashed line areas contain highly eosinophilic cells. Scale bars, 200 µm (left panels), 40 µm (right panels). (D) Sirius Red staining of 12-month-old control, Tab2^HepKO^, and Tak1^HepKO^ liver sections. Scale bars, 200 µm. (E) Fibrosis marker mRNA levels were determined by real-time PCR. Wild type mice, n = 6; Tab2^HepKO^ mice, n = 3. Means ± SEM and P values are shown. (F) 2- to 3-month-old *Tab2*-deficient and control mice were treated with 10 mg/kg mouse weight LPS for 24 h. Protein extracts from the liver were subjected to caspase 3 assay. Wild type mice, n = 5; Tab2^HepKO^ mice, n = 7. Means ± SEM and P values are shown.

**Figure 4:**
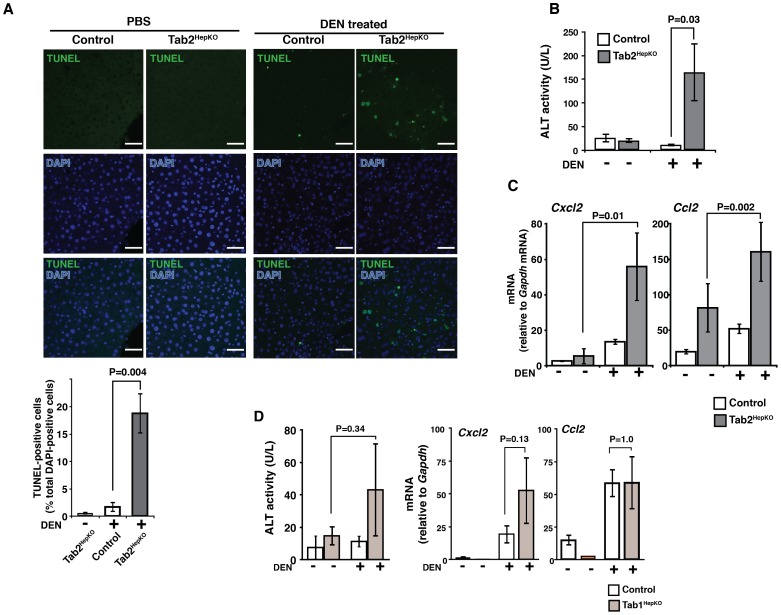
Hepatocyte-specific deletion of Tab2 sensitizes the liver to diethylnitrosamine (DEN)-induced liver injury. (A) 2- to 4-month-old Tab2^HepKO^ and control mice were treated with 100 mg/kg DEN or vehicle (PBS) for 24 h. Liver sections were analyzed by TUNEL staining. Scale bars, 50 µm. TUNEL-positive cells per at least 2000 total liver cells per sample were counted (a graph shown below TUNEL image panels). Controls including wild type and Tab2^HepHet^ mice, n = 4; Tab2^HepKO^ mice, n = 4. Means ± SEM and P values are shown. (B) ALT assay. Controls including wild type and Tab2^HepHet^ mice, n = 7; Tab2^HepKO^ mice, n = 5. Means ± SEM and P values are shown. (C) Expression levels of inflammatory chemokines, *Cxcl2* and *Ccl2*, were determined by real time PCR. Controls including wild type and Tab2^HepHet^ mice, n = 24; Tab2^HepKO^ mice, n = 12. Means ± SEM and P values are shown. (D) Liver from 2- to 4-month-old controls, *Tab1* ^flox/flox^ and *Alb-Cre Tab1* ^flox/+^ and Tab1^HepKO^ mice were analyzed. ALT assay. Control mice, n = 6; Tab1^HepKO^ mice, n = 11. Expression levels of inflammatory chemokines, *Cxcl2* and *Ccl2*, mRNA levels were determined by real time PCR. Controls, mice, n = 4; Tab1^HepKO^ mice, n = 4. Means ± SEM and P values are shown.

To examine whether hepatocyte-derived TAB2 is responsible for the protection against stress-induced liver injury, we generated hepatocyte-specific *Tab2*-deficient (Tab2^HepKO^) mice by using hepatocyte-specific deleter, *alb.Cre*, transgenic mice [33]. The *Tab2* expression in the liver but not lung, brain or heart extract was greatly diminished in the Tab2^HepKO^ mice (Fig. 3A). We also generated Tak1^HepKO^ mice, which are known to develop hepatocellular carcinoma [19], [20]. Tak1^HepKO^ mice exhibited signs of highly increased liver damage including increased levels of serum aminotransferase (ALT), expression of chemokine (C-X-C motif) ligand 2 (*Cxcl2*), chemokine (C-C motif) ligand 2 (*Ccl2*), fibrosis markers, collagen, type I, α1 (*Col1a1*) and tissue inhibitor of metalloproteinase 1 (*Timp1*) as well as hepatocellular carcinoma marker, H19 gene within one month even in the absence of any exogenous stressors (Fig. S2), consistent with earlier studies. In contrast, Tab2^HepKO^ mice developed normally and did not exhibit increased ALT and only marginal or moderate increases in inflammatory chemokine expression in the absence of exogenous stressors as shown in later in Fig. 4. Tab2^HepKO^ liver was histologically indistinguishable from control littermate liver at least at several months of age (Fig. 3B). The lobular architecture was clear in both control and Tab2^HepKO^ liver, although small foci of immune cell infiltration were occasionally observed in all genotypes (Fig. 3B). Aged Tab2^HepKO^ liver exhibited moderately disorganized lobular architectures with relatively large infiltration foci as well as cytoplasmic eosinophilic materials (dashed line) around 10–12 months old of age, while Tak1^HepKO^ showed severe structural damage including unclear lobular architecture in addition to immune cell infiltrations and profound increase of eosinophilic materials (Fig. 3C). Aged Tab2^HepKO^ liver exhibited signs of fibrosis including increased Sirius Red staining, although it is much milder than that in Tak1^HepKO^ (Fig. 3D). Aged Tab2^HepKO^ liver expressed increased fibrosis marker genes, *Col1a1* and *Timp1* (Fig. 3E). These suggest that TAK1 basal activity but not TAB2-dependent TAK1 activation is required for liver integrity at least for several months, and that TAB2 may be important for long-term liver integrity by preventing accumulation of stress-induced liver lesions. To test acute stress-induced liver injury, Tab2^HepKO^ mice were treated with LPS for 24 h. Caspase activity was upregulated in Tab2^HepKO^ liver compared to control mice at 24 h post LPS injection (Fig. 3F); however, the levels of caspase activation were lower compared with those in the ubiquitous *Tab2*-deficient liver described above (see Fig. 2B). This suggests that *Tab2* deficiency highly elevates liver injury also through cell types other than hepatocytes, but that hepatocyte-derived TAB2 is still at least in part responsible for liver protection under the LPS-induced stress condition.

### Hepatocyte-Specific Deletion of Tab2 Sensitizes Mice to Diethylnitrosamine (DEN)-Induced Liver Injury

To further determine the hepatocyte-specific role of TAB2 in stress protection, we utilized a strong liver carcinogen diethylnitrosamine (DEN), which is activated by cytochrome P450 within hepatocytes and induces oxidative stress [39], [40]. DEN-induced stress causes hepatocellular carcinoma in the long term, and acute liver injury in the short term [41]. Tab2^HepKO^ and control mice were treated with DEN for 24 h. We observed highly increased TUNEL-positive cells in Tab2^HepKO^ liver (Fig. 4A) and elevation of serum ALT (Fig. 4B), indicating profound liver injury. Furthermore, the expression of inflammatory genes, *Cxcl2* and *Ccl2*, was elevated in Tab2^HepKO^ liver (Fig. 4C). These results show that hepatocyte-derived TAB2 is required for liver protection from DEN-derived stress. As described earlier, *Tab2* deletion alone in primary hepatocytes diminished TAK1 activation upon LPS challenge (Fig. 1C). However, other stressors may also utilize the TAB1-dependent pathway to activate the TAK1-cell protection pathway. We then examined whether *Tab1*-deficiency sensitizes the liver to DEN. To this end, we generated hepatocyte-specific *Tab1*-deficient (Tab1^HepKO^) mice using the same system as Tab2^HepKO^. Tab1^HepKO^ mice did not exhibit any overt abnormality. Tab1^HepKO^ mice were treated with DEN for 24 h, but did not exhibit statistically significant increases in ALT or in the expression levels of *Cxcl2* and *Ccl2* (Fig. 4E). Thus, TAB1 minimally, if any, contributes to liver protection, whereas TAB2 is indispensable for the protection against stress-induced liver injury.

## Discussion

**Figure 5:**
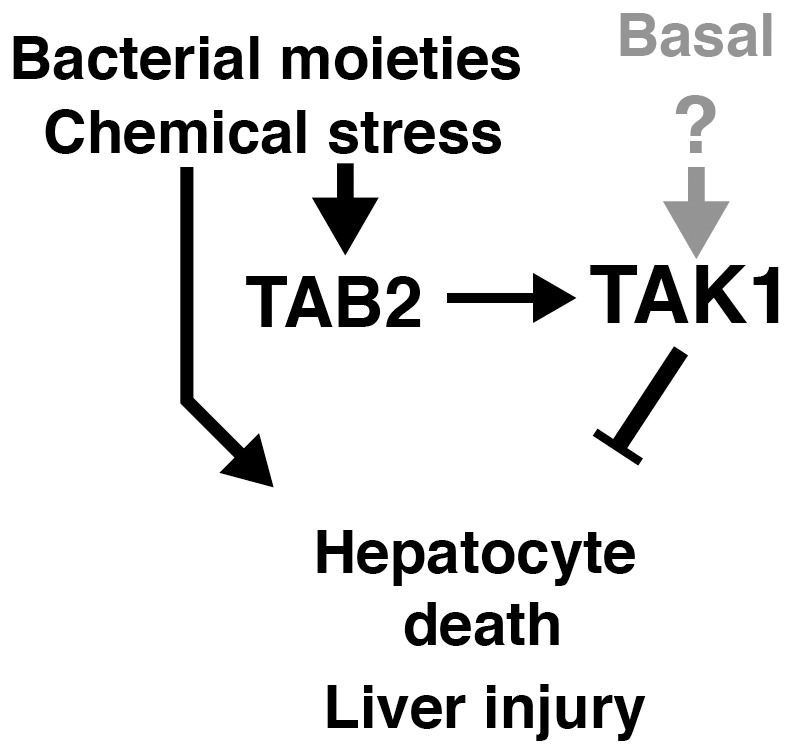
Model.

*Tak1* deficiency in hepatocytes causes spontaneous liver injury which further engages hepatocellular carcinoma development [19], [20]. A previous study in *Tak1* deficient liver used a mouse model that expresses a truncated version of TAK1, which has 38 amino acids deleted around the ATP binding site of the protein kinase domain but the other domains intact [19], [30]. Thus, TAK1 kinase activity but not TAK1 protein itself is important for hepatocyte survival. Accordingly, it is reasonable to assume that TAK1 is active to some extent in the liver under homeostatic conditions, and constitutively upregulates pro-survival factors such as cIAP and c-Jun [15], [16], and anti-oxidants such as Nrf2 and NAD(P)H:quinone oxidoreductase 1 [18], [42]. Earlier studies have established that this TAK1 pro-survival signaling pathway is indispensable for liver protection [19], [20]. In the current study, we found that a TAK1 activator protein, TAB2, is indispensable for stressor-induced TAK1 activation in hepatocytes, and that TAB2 is required for prevention of hepatocyte death in response to stressor challenge *in vivo*. We propose that TAK1 activity is required for prevention of hepatocyte cell death under basal conditions, and that, under stressed conditions, TAK1 is activated beyond the basal level through TAB2, which is required for liver protection against stressors as summarized in Fig. 5.

As shown in Fig. 3, hepatocyte-specific *Tab2* deletion did not cause any abnormality at least for several months, whereas *Tak1* deficiency induced profound liver injury and even tumors within 4 weeks of age (Fig. S2) in agreement with the earlier studies [19], [20]. Thus, TAB2 is not required for the basal TAK1 activity to protect hepatocytes under the unstressed condition. TAB2 is known to play a major role in cytokine and Toll-like receptor (TLR)-induced TAK1 activation through a polyubiquitin-dependent mechanism [24]. Hence, the cytokine- or TLR ligand-induced pathway is not essential for basal hepatic TAK1 activity. How is TAK1 activated in the absence of TAB2? Another TAK1 binding partner, TAB1, can activate TAK1 through promoting oligomerization of TAK1 [13], [21]. TAB1 has been identified to be essential for maintenance of TAK1 activity in the epidermis [28]. We show that *Tab1* deficiency greatly reduces TAK1 activity in the epidermis. TAB1 may be responsible for the basal TAK1 activity in the liver, too. However, *Tab1* deletion alone does not cause any abnormalities in the liver. Thus, *Tab1* is not essential as is *Tab2* for hepatocyte survival *in vivo*. TAB3 can mediate polyubiquitin-dependent activation of TAK1, but *Tab3* deletion alone does not cause any abnormalities during embryogenesis or in adult mice [23]. Collectively, it is likely that TAB1 and TAB2 and possibly TAB3 redundantly function to activate TAK1 under the basal conditions. Thus, single deletion of *Tab1*, *Tab2* or *Tab3* does not cause spontaneous liver injury.

Blockade of TAK1 signaling is a powerful method to kill cells *in vivo*. However, since the TAK1-mediated cell survival pathway is indispensable for liver integrity, TAK1 is not an ideal target to remove undesired hepatocytes such as tumor cells. *Tak1* deletion even causes hepatocellular carcinoma [19], [20]. *Tab2* deficiency alone does not cause any abnormality in unstressed liver at least for several months in the mouse model. Therefore, inhibition of TAB2 would not cause acute liver injury in normal liver. Our results suggest that TAB2-dependent activation of TAK1 beyond the basal level is required for hepatocyte survival only when hepatocytes are under stressed conditions. Thus, we anticipate that inhibition of TAB2 might selectively kill stressed hepatocytes such as hepatocellular carcinoma cells. TAB2 could be a new target to kill stressed or damaged hepatocytes without affecting unstressed cells.

## Supporting Information

Figure S1 **LPS-induced NF-κB activation is reduced by** ***Tak1*** **or** ***Tab2*** **deficiency in primary hepatocytes.**
(TIF)

Figure S2 ***Tak1*** **deficiency induces spontaneous liver injury within one month.**
(TIF)
