# Supplementary figures and images for: TAK1 Binding Protein 2 Is Essential for Liver Protection from Stressors
Source: PLoS One. 2014 Feb 3;9(2):e88037. doi: 10.1371/journal.pone.0088037 (PMC3912198; doi:10.1371/journal.pone.0088037)

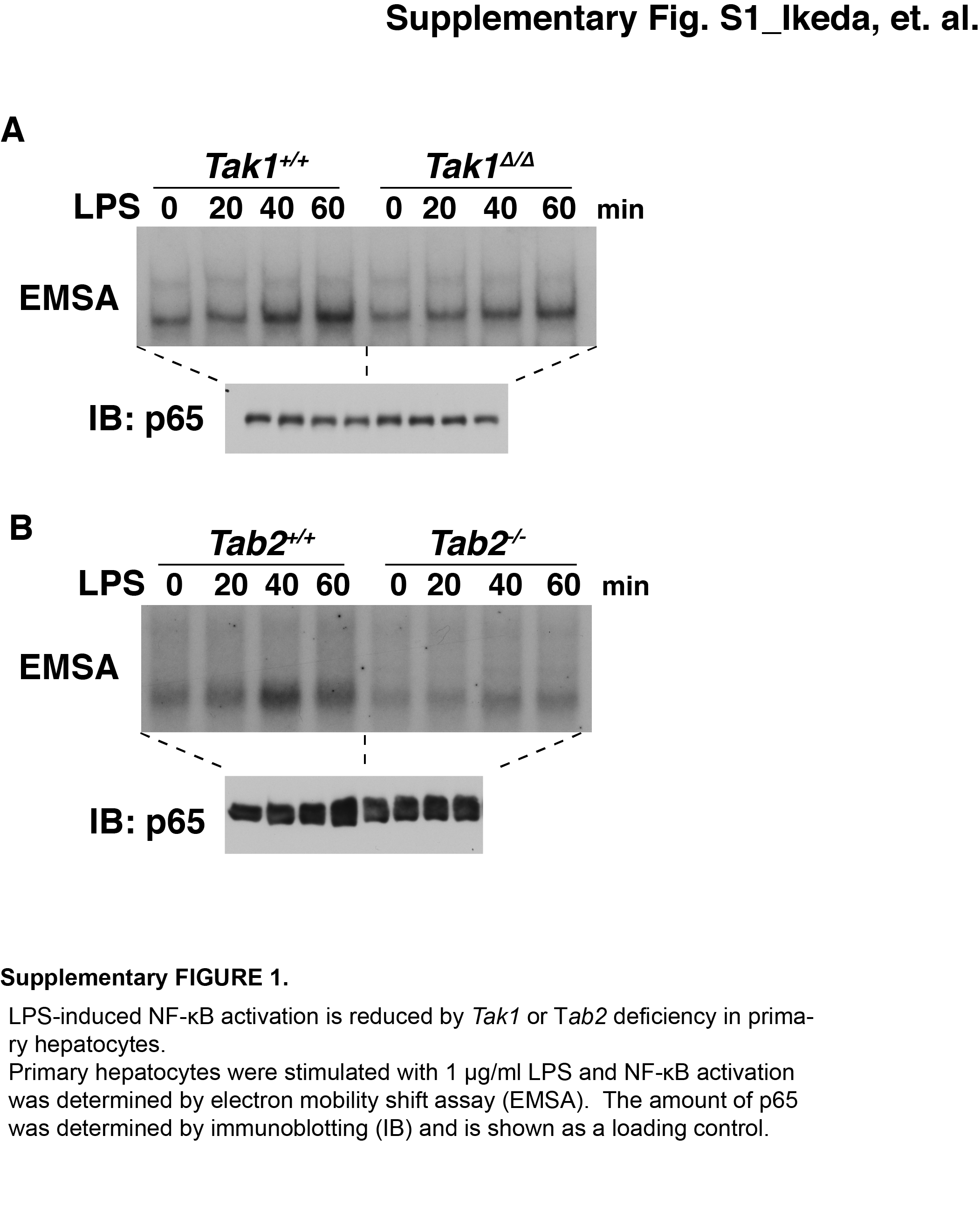

Supplement: Figure S1 — LPS-induced NF-κB activation is reduced by Tak1 or Tab2 deficiency in primary hepatocytes. (TIF) [file pone.0088037.s001.tif]

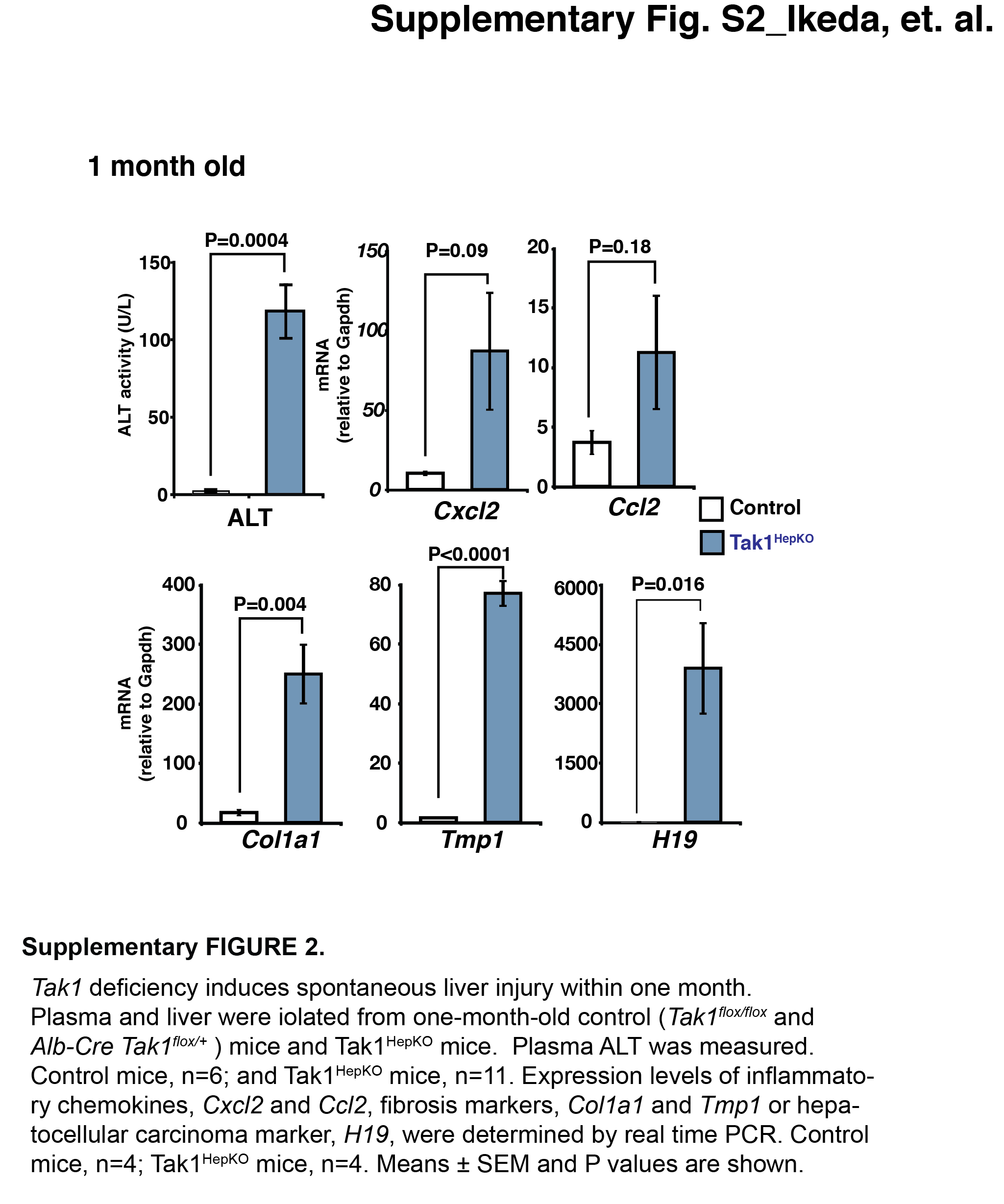

Supplement: Figure S2 — Tak1 deficiency induces spontaneous liver injury within one month. (TIF) [file pone.0088037.s002.tif]
